# How Deep Learning in Antiviral Molecular Profiling Identified Anti-SARS-CoV-2 Inhibitors

**DOI:** 10.3390/biomedicines11123134

**Published:** 2023-11-24

**Authors:** Mohammed Ali, In Ho Park, Junebeom Kim, Gwanghee Kim, Jooyeon Oh, Jin Sun You, Jieun Kim, Jeon-Soo Shin, Sang Sun Yoon

**Affiliations:** 1 Department of Microbiology and Immunology, Yonsei University College of Medicine, Seoul 03722, Republic of Korea; midoabdo@yonsei.ac.kr (M.A.); june0595@yonsei.ac.kr (J.K.); kgh0531@gmail.com (G.K.); jyeon3538@naver.com (J.O.); jinsunyou@naver.com (J.S.Y.); jieunkim20199@yuhs.ac (J.K.); 2 Brain Korea 21 Project for Medical Sciences, Department of Microbiology and Immunology, Yonsei University College of Medicine, Seoul 03722, Republic of Korea; 3 Department of Biomedical Science, Yonsei University College of Medicine, Seoul 03722, Republic of Korea; inhopark@yuhs.ac; 4 Institute of Immunology and Immunological Diseases, Yonsei University College of Medicine, Seoul 03722, Republic of Korea; 5 BioMe Inc., Seoul 02455, Republic of Korea

**Keywords:** SARS-CoV-2, artificial intelligence, compounds library, nucleoside analogs, azathioprine, thioinosinic acid

## Abstract

The integration of artificial intelligence (AI) into drug discovery has markedly advanced the search for effective therapeutics. In our study, we employed a comprehensive computational–experimental approach to identify potential anti-SARS-CoV-2 compounds. We developed a predictive model to assess the activities of compounds based on their structural features. This model screened a library of approximately 700,000 compounds, culminating in the selection of the top 100 candidates for experimental validation. In vitro assays on human intestinal epithelial cells (Caco-2) revealed that 19 of these compounds exhibited inhibitory activity. Notably, eight compounds demonstrated dose-dependent activity in Vero cell lines, with half-maximal effective concentration (EC50) values ranging from 1 μM to 7 μM. Furthermore, we utilized a clustering approach to pinpoint potential nucleoside analog inhibitors, leading to the discovery of two promising candidates: azathioprine and its metabolite, thioinosinic acid. Both compounds showed in vitro activity against SARS-CoV-2, with thioinosinic acid also significantly reducing viral loads in mouse lungs. These findings underscore the utility of AI in accelerating drug discovery processes.

## 1. Introduction

The COVID-19 pandemic, triggered by the SARS-CoV-2 virus, has had a profound global impact, resulting in millions of infections and a staggering death toll. This crisis has necessitated an urgent search for effective treatments to reduce illness severity, prevent complications, and curb viral transmission [1,2]. The development of new therapies is crucial not only for managing the current pandemic but also in preparation for future coronavirus outbreaks.

SARS-CoV-2, a positive-sense, single-stranded RNA virus, primarily spreads via respiratory droplets. Its life cycle includes key stages such as binding to host cell receptors (mainly *ACE-2*), entry into cells, genome replication, viral protein synthesis, virion assembly, and release to infect additional cells. Essential viral proteins such as the spike protein, main protease, and replicase complex (including *RdRp*) are critical for the virus’s replication and spread [3,4,5,6]. A detailed understanding of these molecular interactions is vital for developing effective SARS-CoV-2 treatments [7,8].

Despite the significant impact of COVID-19, effective treatments for SARS-CoV-2 remain limited. Current strategies focus on supportive care, with antivirals like remdesivir showing some effectiveness in reducing hospital stay duration but having limited impact on overall clinical outcomes [9,10,11,12]. Molnupiravir has shown promise in impeding viral replication by introducing genetic mutations, though concerns about the spread of mutation-bearing viral particles persist [13]. Paxlovid, a combination of nirmatrelvir and ritonavir, has been effective but is not recommended for patients with renal or hepatic impairments [14]. Ensitrelvir, targeting the viral 3CL protease, has shown encouraging results in early studies, but its efficacy against newer variants remains unverified [15]. Corticosteroids like dexamethasone have reduced mortality in severe cases, but their use is contentious [16,17]. Hence, there is a continued emphasis on discovering novel, effective therapies.

The drug discovery process, traditionally reliant on experimental methods, is being revolutionized by artificial intelligence (AI). AI enhances drug discovery by predicting potential drug’s binding affinities to target proteins, identifying promising drug candidates through analysis of protein–ligand interaction datasets [18,19,20,21]. AI also plays a pivotal role in drug repurposing, predicting interactions of existing drugs with various proteins, thereby uncovering new therapeutic applications [22,23,24,25]. Additionally, AI can optimize clinical trials by predicting patient responses and effective drug dosages, potentially reducing trial duration and costs [26,27].

AI success in drug discovery is contingent on access to extensive, high-quality datasets. The COVID-19 pandemic has seen unprecedented collaboration in data sharing among pharmaceutical companies and academic institutions, enriching the datasets available for AI algorithms [28,29,30]. The National Center for Advancing Translational Sciences (NCATS) has notably launched the SARS-CoV-2 Open Data Portal, consolidating extensive datasets for AI analysis [31]. Past applications of AI have successfully identified SARS-CoV-2 inhibitors [32,33,34,35,36] and proposed novel drug combinations [37], demonstrating the feasibility of rapid antiviral compound development [38].

Our study embarked on a comprehensive search for potential SARS-CoV-2 treatments, integrating AI-driven computational screening with experimental validation using in vitro and in vivo models. This approach led to the identification of several compounds with potent antiviral activity against SARS-CoV-2, including azathioprine, an existing FDA-approved medication, as a promising candidate for inhibiting viral replication.

## 2. Materials and Methods

### 2.1. Data Preparation

Our predictive model was developed using data curated from the COVID-19 NCATS repository. This repository encompasses a diverse range of compounds, including those that are approved, investigational, bioactive, and natural—all screened for their activity against SARS-CoV-2. For the training dataset, we meticulously selected 100 drugs known for their potent antiviral properties and low toxicity profile in the Vero cell line. Additionally, over 2000 compounds demonstrating no inhibitory activity against SARS-CoV-2 were included to enhance the model’s robustness. Following the initial training phase, the model was tasked with analyzing a new dataset comprising approximately 700,000 compounds sourced from the KCB database. This step aimed to identify potential inhibitors of SARS-CoV-2.

To further refine the model’s accuracy and predictive capabilities, we integrated a subset of nucleoside analogs, including remdesivir and molnupiravir, along with their parent compounds. These additions were particularly strategic, as these drugs have established efficacy against SARS-CoV-2.

### 2.2. Model

For the identification of potential antiviral compounds against SARS-CoV-2, we utilized DeepChem, an open-source drug discovery platform, to develop a classification model. Our model was trained on a dataset, comprising molecules represented by Simplified Molecular Input Line Entry System (SMILES) strings. These molecules were categorized with corresponding labels: active drugs were labeled as ‘1’ and inactive compounds, as ‘0’.

To discern patterns within the training data, we employed a graph convolutional network (GCN). In this approach, each molecule is conceptualized as a graph. The GCN processes the chemical properties of individual atoms and assimilates information from their interconnectedness, thereby generating a new data vector for each atom. Through successive GCN layers, the atomic embeddings are aggregated to construct a comprehensive representation of the entire molecule. This molecular representation is then input into a fully connected layer, which predicts the molecule’s potential antiviral activity.

To ensure robust generalization of new, unseen data, we partitioned the dataset into two portions: 80% for training and 20% for testing. The model demonstrated notable predictive proficiency, achieving a high Receiver Operating Characteristic–Area Under Curve (ROC-AUC) score of 0.85 on the test dataset.

### 2.3. Compound Similarities

To evaluate the similarity among active compounds in our training dataset, we utilized the Tanimoto similarity metric, a standard method in cheminformatics. For this purpose, we employed the RDKit library to generate molecular fingerprints of each compound. These fingerprints, expressed as binary values, signify the presence or absence of predefined substructures within the compounds. By comparing these molecular fingerprints, we calculated similarity scores for each compound pair, with scores ranging from 0 (no similarity) to 1 (identical). To facilitate an intuitive visualization of these similarities, we created a heatmap using the Matplotlib and Seaborn libraries. This graphical representation provides a clear, easily interpretable depiction of the relational structure among various compounds.

Furthermore, we extended our analysis using t-SNE plots, which were generated through the Chemplot Python library. This technique allowed us to cluster compounds based on their structural similarities. Notably, within these clusters, we identified compounds predicted by KCB that were structurally proximal to known nucleoside analogs, offering further insights into potential antiviral properties.

### 2.4. Cells and Virus

We cultivated three cell lines: Caco-2 epithelial cells (ATCC HTB-37), Calu-3 cells (ATCC HTB-55), and Vero E6 cells (ATCC CRL-1586), using Dulbecco’s Modified Eagle Medium (DMEM) supplemented with 10% fetal bovine serum and 1% penicillin/streptomycin. These cell cultures were maintained in an incubator set to a controlled environment of 37 °C and 5% CO2, ensuring optimal growth conditions.

For the virus studies, we procured SARS-CoV-2 and its variants from the National Culture Collection for Pathogens (NCCP) at the Korea Disease Control and Prevention Agency (KDCA) in Osong, Korea. The virus strains used included the B.1 (NCCP43326, wild type) and B.1.351 (NCCP43382, beta variant). To amplify these viruses, we infected Vero E6 cells and subsequently assessed viral infectivity using a plaque assay. This assay enabled us to quantify the infectious units of the SARS-CoV-2 virus.

### 2.5. ELISpot Assay/Focus Reduction Neutralization Test (FRNT)

For the ELISpot assay, Vero cells were cultured in 96-well plates (Corning #3596, Tewksbury, MA, USA) at a density of 2 × 10^4^ cells per well for a duration of 24 h. The drugs under investigation were serially diluted twofold in DMEM media supplemented with 2% FBS. After removing the original media, the cells were treated with 50 µL of these drug solutions and incubated for 2 h prior to SARS-CoV-2 virus infection. The virus was introduced at a multiplicity of infection (MOI) of 0.1 and allowed to incubate for 8–10 h. Post-infection, the cells were fixed in 4% paraformaldehyde overnight and subsequently permeabilized with cold methanol for 15 min. For antibody staining, the cells were washed with PBS and blocked for 30 min using 5% calf serum. The cells were then incubated with a primary antibody targeting the SARS-CoV-2 nucleocapsid protein (Sino Biological, Beijing, China) for 1 h, followed by three PBST washes. This was succeeded by incubation with a horseradish peroxidase (HRP)-conjugated secondary goat anti-rabbit IgG antibody (Jackson ImmunoResearch Laboratories, West Grove, PA, USA), and another set of three PBST washes. Subsequently, the cells were treated with KPL TrueBlue™ peroxidase substrate (SeraCare, Milford, MA, USA) for 5 min. After a final wash with water, cell foci were imaged and quantified using an ImmunoSpot^®^ S6 Micro Analyzer (CTL—Europe GmbH, Bonn, Germany). The half-maximal effective concentration (EC50) of each drug was calculated as the reciprocal of the last dilution, resulting in 50% foci reduction compared to control wells (no drug inhibitor). EC50 values were interpolated using nonlinear regression (curve fitting) in GraphPad Prism v.9 (GraphPad Software, San Diego, CA, USA). 

### 2.6. Immunofluorescence Assay

For the immunofluorescence assay, Calu-3 cells were seeded in a 96-well plate at a density of 3 × 10^4^ cells per well and incubated overnight to facilitate adherence and growth. The following day, the media was removed, and the cells were gently washed with Dulbecco’s Phosphate-Buffered Saline (DPBS). This step was followed by the addition of 50 µL of each drug to the wells, achieving a final concentration of 5 µM. After a 2 h incubation period with the drugs, the cells were infected with SARS-CoV-2 at a multiplicity of infection (MOI) of 0.1 and incubated for 72 h. Post-infection, the cells were fixed with 4% paraformaldehyde overnight. For antibody staining, the cells were first permeabilized with cold methanol for 15 min. Following this, they were washed with PBST and blocked for 30 min using 5% calf serum. To detect viral presence, the cells were incubated with a primary anti-SARS-CoV-2 nucleocapsid antibody for 1 h. After three PBST washes, the cells were incubated with a secondary goat anti-rabbit IgG antibody conjugated with Alexa Fluor 488 (Thermo Fisher Scientific, Waltham, MA, USA). Subsequent to three additional PBST washes, fluorescence signals were detected using a fluorescence reader, employing an excitation wavelength of 488 nm. 

### 2.7. Virus Inhibition Assay

To assess the inhibitory effects of drugs on SARS-CoV-2, we conducted a virus inhibition assay using Calu-3 cells. Initially, Calu-3 cells were seeded at a density of 3 × 10^4^ cells per well in 96-well plates and cultured overnight. Following this incubation period, the media was discarded, and the cells were gently rinsed with Dulbecco’s Phosphate-Buffered Saline (DPBS). Subsequently, the cells were infected with SARS-CoV-2 at a multiplicity of infection (MOI) of 0.1. This infection was allowed to proceed for 1 h, after which the cells were again washed with DPBS to remove unbound virus particles. Post-infection, the cells were treated with varying concentrations of the drugs, ranging from 0.6 µM to 10 µM, diluted in media containing 2% FBS. After a 72 h incubation period, the supernatants from the cell cultures were collected. These supernatants were then subjected to plaque assays to quantify viral infectivity, providing crucial insights into the effectiveness of the drug treatments in inhibiting SARS-CoV-2 replication.

### 2.8. Plaque Assay

For the plaque assay, Vero E6 cells were plated at a density of 3 × 10^5^ cells per well in a 12-well plate and incubated for 24 h to allow for cell adherence and growth. Following this incubation period, the growth medium was removed, and the cells were washed with Dulbecco’s Phosphate-Buffered Saline (DPBS). To initiate infection, 200 µL of virus-containing supernatants, diluted tenfold, were applied to each well. The cells were then incubated for 1 h to facilitate virus entry. After this incubation, the virus-containing solution was discarded, and a mixture of 2× DMEM and 2% low-melting-point agar solution at a 1:1 ratio was added to each well. This overlay medium served to immobilize the virus and limit its spread. The cells were subsequently incubated for an additional 3–4 days, a duration optimized for the development of visible virus plaques. Upon plaque formation, the cells were stained using a solution of 0.5% crystal violet in 20% methanol. This staining allowed for the clear visualization and enumeration of virus plaques, facilitating the quantitative analysis of viral infectivity.

### 2.9. Quantification of Virus Protein

To quantify viral protein levels, Calu-3 cells were plated at a density of 1 × 10^5^ cells per well in a 48-well plate and cultured overnight. The following day, these cells were infected with SARS-CoV-2 at a multiplicity of infection (MOI) of 0.1 for 1 h. After removing the virus, the cells were treated with various drug concentrations for 72 h in fresh media. For protein extraction, the infected cells were lysed using 50 µL of RIPA cell lysis buffer (GenDEPOT, Baker, TX, USA). The lysed cells were then subjected to sonication in three 6 min cycles to ensure complete disruption. The lysates were centrifuged at 15,000 rpm for 20 min, allowing for the collection of supernatants containing the proteins of interest. The protein content in these supernatants was quantified using the bicinchoninic acid (BCA) assay. Subsequently, protein samples were separated via 10% SDS-PAGE gel electrophoresis and transferred onto a nitrocellulose membrane for further analysis. To detect specific viral proteins, the membrane was first blocked with 5% skim milk for 30 min to prevent nonspecific binding. It was then incubated overnight at 4 °C with a primary antibody targeting the SARS-CoV-2 nucleocapsid protein (dilution 1:3000). Following this, the membrane was incubated for 1 h with a horseradish peroxidase (HRP)-conjugated secondary antibody (dilution 1:3000). Finally, the viral proteins were visualized using a chemiluminescent solution.

### 2.10. Drug Cytotoxicity Assays

To assess the cytotoxicity of the drugs under investigation, we conducted an assay using Vero cells. Initially, the cells were seeded in a 96-well plate at a density of 1 × 10^4^ cells per well and cultured for 24 h to ensure proper adherence and growth. Subsequently, the culture medium was discarded, and the cells were exposed to various concentrations of the drugs. These drugs were diluted in DMEM media supplemented with 2% FBS. Following a 72 h incubation period with the drugs, the extent of cellular toxicity was evaluated using the Cell Counting Kit-8 (CCK-8) assay kit (Dojindo, Kumamoto, Japan). This assay is based on the reduction of a water-soluble tetrazolium salt by cellular dehydrogenases in viable cells, producing a colorimetric change that can be quantified. The intensity of the color change is directly proportional to the number of living cells, thus providing an indication of the cytotoxic effects of the drugs.

### 2.11. Animal Experiments

In vivo experiments were conducted using 7-week-old female BALB/C mice, which were housed under sterile conditions with access to food and water in a Biosafety Level 3 (BSL-3) facility at the Department of Laboratory Animals, Avison Biomedical Research Center, Yonsei University College of Medicine. This study received approval from the Institutional Animal Care and Use Committee at Yonsei University College of Medicine (permit number: 2022-0228) and was carried out in strict adherence to the relevant guidelines and regulations. Furthermore, this study conforms to the ARRIVE guidelines for reporting in vivo experiments.

For the infection protocol, the mice were anesthetized and intranasally inoculated with 1 × 10^5^ plaque-forming units (pfu) of the beta variant of SARS-CoV-2, administered in a volume of 50 µL. Two hours post-infection, the mice were treated with either 10% DMSO as a vehicle control or with therapeutic doses of azathioprine (5 mg/kg) or thioinosinic acid (5 mg/kg). A subsequent dose of the drugs was administered one day after the initial infection. The mice were monitored daily for changes in body weight until four days post-infection. At this endpoint, the mice were humanely sacrificed, and their lungs were collected for subsequent analysis. Virus titers in the lung tissues were quantified using the plaque assay method, providing insights into the efficacy of the treatments in reducing viral loads in vivo.

### 2.12. Statistical Analysis

The data in this study are presented as means ± standard deviation (S.D.). We employed GraphPad Prism version 9 (GraphPad Software Inc., La Jolla, CA) for all data calculations and statistical analyses. To assess the significance of differences between experimental groups, we used a one-way analysis of variance (ANOVA), and *p* values < 0.05 were considered statistically significant. * *p* < 0.05; ** *p* < 0.01; *** *p* < 0.001; **** *p* < 0.0001.

## 3. Results

### 3.1. Computational Screening of Potential Anti-SARS-CoV-2 Inhibitors

**Figure 1:**
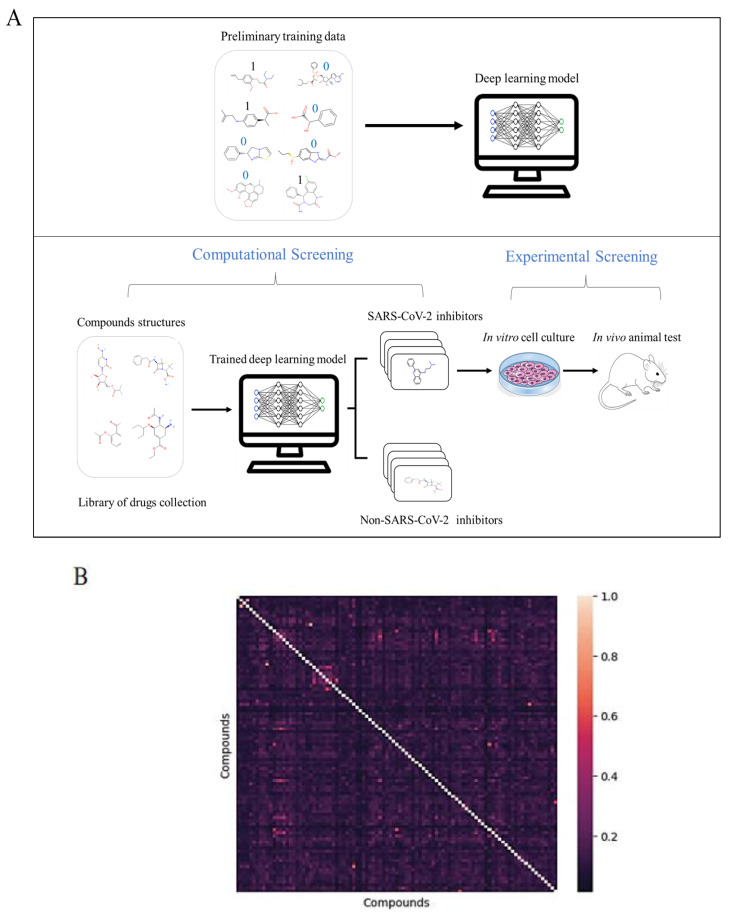
Overview of computational and experimental screening for SARS-CoV-2 inhibitors. (**A**) This flowchart delineates the sequential stages involved in identifying potential anti-SARS-CoV-2 compounds. The initial stage encompasses the collection of compound structures and their corresponding activity labels (with ‘1’ indicating active and ‘0’ indicating inactive compounds). Subsequently, these compounds are subjected to a training phase using a graph neural network, which analyzes their structural and functional characteristics. In the inference stage, the trained model is applied to the Korea Chemical Bank (KCB) drug collection to identify promising hit compounds. The final stage involves experimental validation of selected compounds, conducted in various cell lines and a mouse disease model. (**B**) Heatmap of structural co-similarity: This heatmap presents the structural co-similarity matrix of the compounds, calculated using the Tanimoto similarity score. The x and y axes of the heatmap list the active compounds in the dataset. The diagonal line represents the self-similarity of each compound. The color gradient in the heatmap conveys the degree of similarity between compounds: darker colors signify lower similarity, while lighter colors indicate higher similarity.

Our initial step in identifying potential antiviral agents involved establishing a robust computational pipeline. We utilized raw data from previous experimental screenings of SARS-CoV-2, accessible through the COVID-19 Open Data Portal by the National Center for Advancing Translational Sciences (NCATS). This dataset included assessments of the cytopathic effect of SARS-CoV-2 on the Vero E6 cell line. From this dataset, we meticulously selected approximately 100 compounds demonstrating high antiviral activity and minimal toxicity to the host cells (Figure 1B and Figure S1). Additionally, we included compounds that showed no activity against SARS-CoV-2 for a comprehensive analysis.

We then developed a deep learning-based classification model using the chemical structures and activity annotations (labeled 1 for active and 0 for inactive) of these compounds. This model, trained to map the structural features of compounds to their functional activity, was developed using the DeepChem open-source Python library for drug discovery. We assessed the model’s performance on a separate test dataset, where it achieved a high Receiver Operating Characteristic Curve–Area Under the Curve (ROC-AUC) score of 0.85, demonstrating its predictive capability in identifying antiviral compounds against SARS-CoV-2 (Figure 1A).

To further our quest for novel antiviral drugs, we applied this trained model to a comprehensive library of approximately 700,000 compounds from the Korea Chemical Bank (KCB). This library includes a wide array of commercially available and proprietary compounds. For each compound, we derived chemical structure data and used our model to predict their potential anti-SARS-CoV-2 activity, assigning probability scores from 0 to 1, with higher scores indicating a higher likelihood of antiviral efficacy.

Our search was then narrowed down to the top 100 compounds predicted by the model. These compounds were physically retrieved from the KCB for subsequent experimental validation across various cell lines.

### 3.2. In Vitro Experimental Validation of Candidate Drug Efficacy

**Figure 2:**
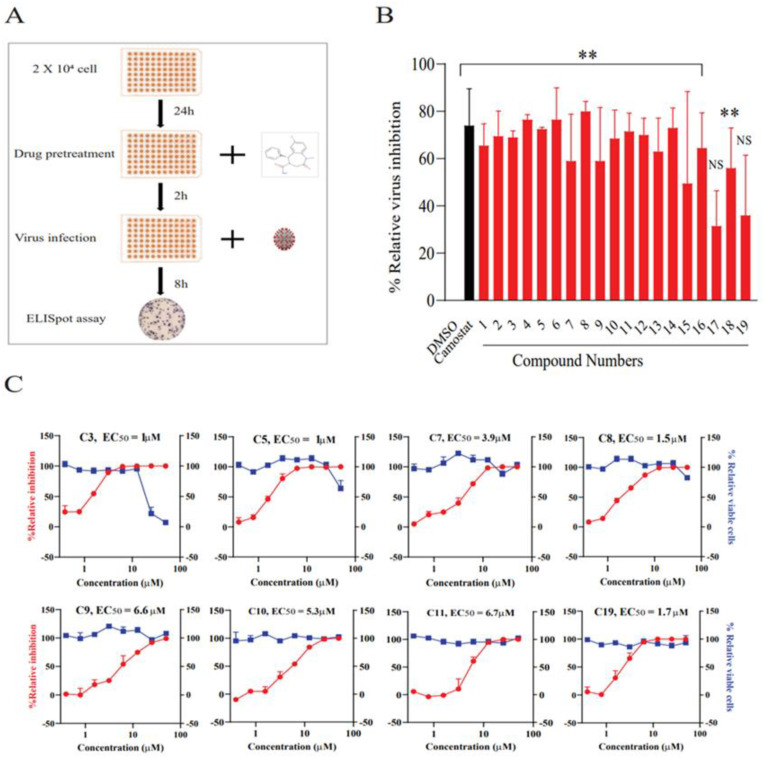
In vitro screening for compounds active against SARS-CoV-2. (**A**) This panel depicts the evaluation of a selected compound’s antiviral activity against SARS-CoV-2 in Caco-2 and Vero cell lines. The cells were pre-treated with the drugs for two hours prior to viral infection. The detection of intracellular virus particles was conducted using the ELISpot assay. (**B**) The primary screening for anti-SARS-CoV-2 activity was performed in Caco-2 cells, which involved pre-treating the cells with a single dose of the test drugs before infection with the virus. Camostat was used as a positive control for this screening. The top active compounds, demonstrating significant inhibitory effects, are highlighted in red in the figure. The data are presented as mean ± standard deviation (SD) for two independent experimental replicates. (**C**) This panel illustrates the dose-dependent response and associated toxicity of the identified hit compounds in the Vero cell line. The level of antiviral activity is indicated in red, showing relative virus inhibition compared to DMSO control samples. The blue color represents cellular toxicity. Data are expressed as mean ± SD for three independent biological replicates. EC50 values were calculated and fitted using GraphPad Prism. Statistical significance was determined using one-way ANOVA with Dunnett’s test, denoted as ** *p* < 0.05. ‘NS’ denotes non-significant findings.

In our in vitro studies, we focused on assessing the antiviral activity of the top 100 compounds shortlisted from the KCB library against SARS-CoV-2. For this purpose, human intestinal epithelial Caco-2 cells [39,40,41,42] were pre-treated with each compound at a fixed concentration of 2.5 µM before viral infection. Post-incubation, we quantified the intracellular virus particles using the ELISpot assay (Figure 2A). Out of the 100 drugs screened, 19 demonstrated significant inhibitory activity in this cell line, achieving up to 80% inhibition. This level of inhibition was notably comparable to that of camostat, a known virus inhibitor that impedes SARS-CoV-2 entry by targeting the TMPRSS2 protease in the host cell (Figure 2B).

To evaluate the concentration-dependent effects of these compounds, we employed Vero cells, which are more susceptible to SARS-CoV-2 infection [43,44,45]. In this assay, cells were treated with varying twofold concentrations of the drugs, ranging from 0.3 µM to 50 µM, prior to viral infection. Among the 19 compounds tested, eight exhibited dose-dependent antiviral activity in Vero cells, with EC50 values ranging between 1 µM and 7 µM. Concurrent cytotoxicity assays revealed that these compounds maintained lower cellular toxicity at effective concentrations, underscoring their potential as therapeutic agents (Figure 2C, Table 1).

### 3.3. Elucidating Molecular Targets of Effective Drugs through Similarity-Based Analysis

In our quest to understand the molecular mechanisms driving the antiviral efficacy of the identified hit compounds, we applied the similarity ensemble approach (SEA) analysis [46]. SEA is a computational method designed to predict the biological activity of compounds based on their structural resemblance to other compounds with known activities. The underlying premise of SEA is that structurally similar molecules are likely to exhibit analogous biological functions. This approach involves scouring a comprehensive database of active compounds, along with their respective biological activities, and calculating similarity scores. These scores are derived by comparing the structural features of our query compounds against the database entries. The calculated similarity scores are then utilized to infer the potential biological activities of the query compounds. From the SEA analysis, we hypothesized that our identified compounds might exert their antiviral effects indirectly, possibly by targeting specific host cell proteins. This inference suggests that these compounds could interfere with critical virus–host interactions or disrupt host cell processes essential for viral replication. Such a mechanism would highlight the potential of these compounds to function as indirect antivirals by modulating host pathways (Table 2).

### 3.4. Identifying Potential Direct-Acting Antivirals through Compounds Clustering

To pinpoint compounds capable of directly inhibiting SARS-CoV-2 replication, we enhanced our deep learning model by incorporating additional examples into our training dataset. This expansion included nucleoside analog inhibitors, such as remdesivir and molnupiravir, and their parent compounds, all known for their direct antiviral activity. Utilizing this augmented model, we performed computational analyses on the Korea Chemical Bank (KCB) dataset to identify potential direct-acting antiviral agents. Given the inclusion of nucleoside analogs in our training dataset, we anticipated that some of the model-predicted compounds might also belong to this class.

**Figure 3:**
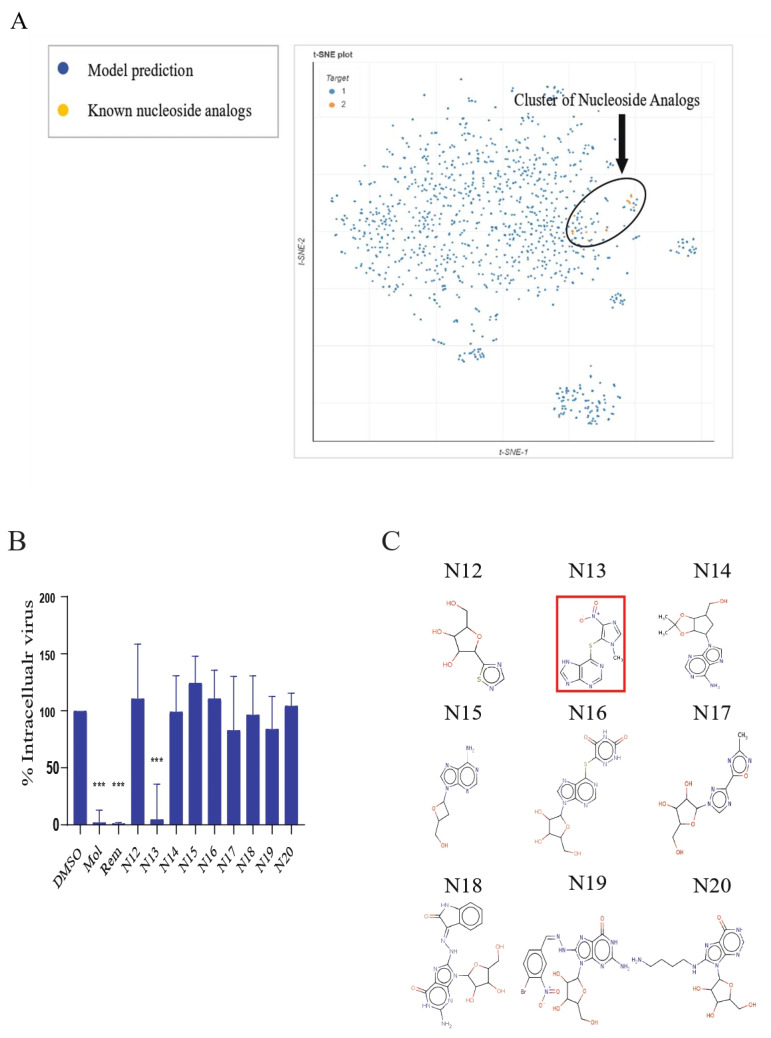
Identification and validation of potential nucleoside analog inhibitors. (**A**) This panel presents a t-SNE plot that demonstrates the clustering of antiviral compounds as predicted by the deep learning model, alongside previously identified nucleoside analogs known to disrupt virus replication. The top 1000 compounds predicted by the model are represented in blue, while the known nucleoside analogs are depicted in orange, illustrating their relative positions in the chemical space. (**B**) The antiviral efficacy of compounds located near the nucleoside analogs cluster was assessed in the Calu-3 cell line. Cells were pre-treated with these compounds (or DMSO as a mock control) for two hours before being infected with SARS-CoV-2 at an MOI of 0.1. The inhibitory effects on viral replication were evaluated after 72 h using an immunofluorescence assay. Established nucleoside analogs, molnupiravir and remdesivir, were employed as controls. The graph shows the percentage of the intracellular fluorescent signal relative to the mock sample, with error bars indicating the mean ± standard deviation (SD) from triplicate biological replicates. Statistical significance was assessed using one-way ANOVA with Dunnett’s test, denoted as *** *p* < 0.001. (**C**) This panel displays the molecular structures of the compounds tested, with azathioprine highlighted in red.

To distinguish potential nucleoside analogs from other compounds, we employed the t-distributed stochastic neighbor embedding (t-SNE) method from the ChemPlot chemical space visualization library [47]. This clustering technique was applied to the top 1000 compounds predicted by our model, along with the original nucleoside analogs used during training. The analysis resulted in the identification of a distinct cluster of nucleoside analogs. Notably, several compounds predicted by the KCB were closely aligned with this cluster, suggesting their potential as nucleoside analog inhibitors (Figure 3A).

To explore the antiviral capabilities of these compounds, we selected nine KCB compounds located near the nucleoside analog cluster for in vitro validation in Calu-3 cells (Figure 3A). The efficacy of these compounds was evaluated by quantifying intracellular virus concentrations using immunofluorescence staining. Remarkably, one of these compounds, azathioprine, demonstrated antiviral activity comparable to established nucleoside analog inhibitors, specifically remdesivir and molnupiravir (Figure 3B,C).

### 3.5. In Vitro Efficacy of Azathioprine and Thioinosinic Acid against SARS-CoV-2

**Figure 4:**
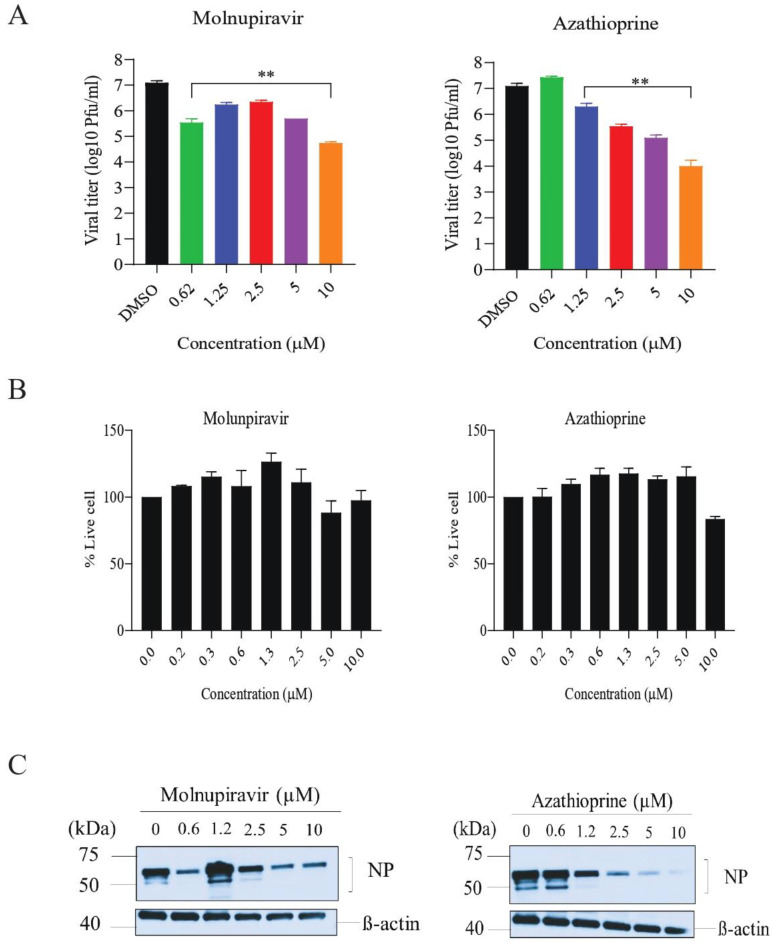
In vitro inhibition of SARS-CoV-2 infection by azathioprine. (**A**) This panel illustrates the inhibitory effect of azathioprine on SARS-CoV-2 in the Calu-3 cell line. The cells were first infected with the virus at a multiplicity of infection (MOI) of 0.1. One hour post-infection, azathioprine was administered at various twofold dilutions and maintained for 72 h. The infectious virus titers in the cell supernatants were quantified using a plaque assay. The error bars represent the mean ± standard deviation (SD) of duplicate samples. Statistical significance was evaluated using one-way ANOVA with Dunnett’s test, indicated by ** *p* < 0.05. (**B**) In this panel, the cytotoxicity of azathioprine in uninfected Calu-3 cells was measured and normalized against cells treated with DMSO. The graph provides a comparison of the cytotoxic effects of azathioprine relative to the DMSO control. (**C**) Calu-3 cell lysates were subjected to Western blot analysis using an anti-SARS-CoV-2 nucleocapsid antibody. An anti-ß-actin antibody was used as a control.

We investigated the dose-dependent antiviral effects of azathioprine on the Calu-3 cell line, a model commonly used in SARS-CoV-2 research [48,49,50]. After infecting the cells with the virus, we treated them with varying concentrations of azathioprine, up to a maximum of 10 µM. As a control, we used molnupiravir, a known virus inhibitor. Our analyses, including plaque assays and Western blotting, indicated a significant reduction in viral progeny within the cells treated with azathioprine (Figure 4A,C), highlighting its potential as a potent antiviral agent against SARS-CoV-2. Furthermore, the low cellular toxicity observed at these concentrations reinforces the drug’s safety profile (Figure 4B). While molnupiravir exhibited moderate inhibitory effects, which we further confirmed using RT-PCR assay (Figure S3), it is important to note that the antiviral efficacy observed may vary depending on the cell line used, given differences in drug uptake and metabolic pathways.

Azathioprine, a prodrug, undergoes metabolic activation in the body. Initially, it converts into 6-mercaptopurine (6-MP) via non-enzymatic reduction, facilitated by glutathione and similar compounds present in the intestinal wall, liver, and red blood cells. This transformation yields the intermediate metabolite thioinosinic acid, which then follows one of two metabolic pathways. In one pathway, it produces thioguanosine triphosphate, contributing to the drug’s therapeutic activity. Alternatively, the compound undergoes methylation and is eventually excreted in the urine [51]. We extended our investigation to examine the direct anti-SARS-CoV-2 effects of thioinosinic acid. Post viral infection, Calu-3 cells were treated with various concentrations of thioinosinic acid. This assessment revealed the notable antiviral efficacy of thioinosinic acid against SARS-CoV-2 in the Calu-3 cell line (Figure S4).

### 3.6. In Vivo Efficacy of Thioinosinic Acid in Reducing SARS-CoV-2 Infection in a Mouse Model

**Figure 5:**
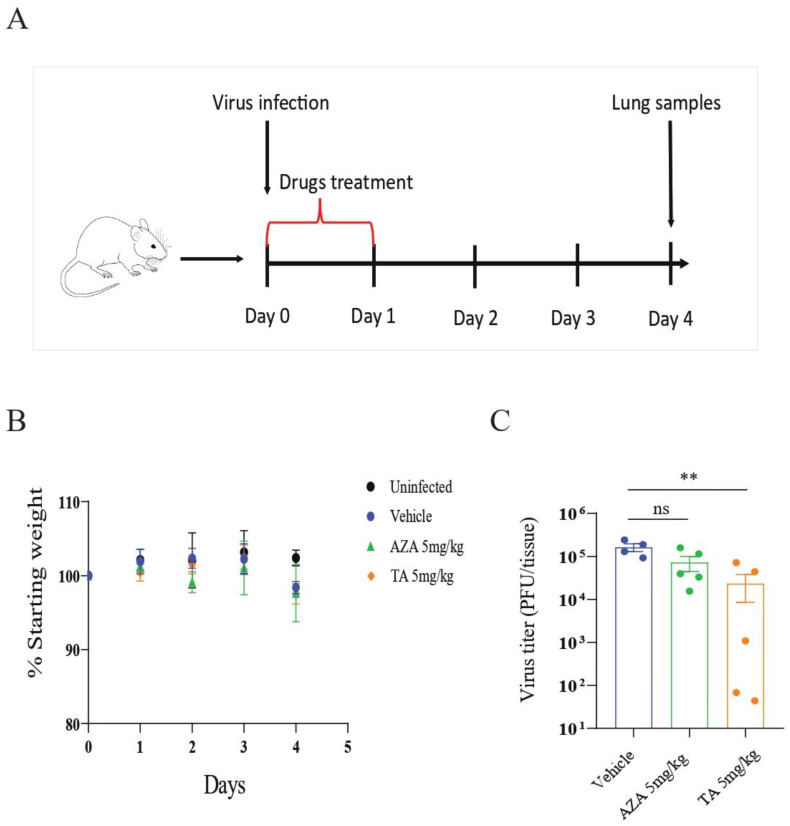
Effect of thioinosinic acid on virus load in a mouse model. (**A**) This panel outlines an in vivo experiment involving the intranasal inoculation of mice with 1 × 10^5^ plaque-forming units (pfu) of the SARS-CoV-2 beta variant. Two hours after inoculation, an intranasal administration of the drugs was initiated, followed by a second dose administered 24 h later. Four days post-inoculation, the mice were euthanized, and their lungs were harvested for subsequent analysis. (**B**) This graph tracks the body weight changes over time for different groups: uninfected mice, shown in black (*n* = 3), infected mice treated with the vehicle, shown in blue (*n* = 4), and infected mice treated with azathioprine (AZA) at 5 mg/kg, shown in green (*n* = 5) or thioinosinic acid (TA) at 5 mg/kg, shown in orange (*n* = 5). (**C**) The viral load in the lung tissues of the mice was quantified using a plaque assay. The error bars represent the mean ± standard deviation (SD) of duplicate samples from each group. Statistical significance was assessed using one-way ANOVA with Dunnett’s test, denoted as ** *p* < 0.005. This panel provides crucial data on the efficacy of the drug treatments in reducing viral titers in the lungs.

To assess the in vivo efficacy of azathioprine and thioinosinic acid against SARS-CoV-2, we conducted an experiment using a mouse model. Mice were infected intranasally with the virus and subsequently administered either azathioprine or thioinosinic acid intranasally, beginning two hours post-infection and followed by a single dose on the subsequent day. A control group was treated with 10% DMSO intranasally as a vehicle control (Figure 5A). Over a four-day observation period, all of the infected mice exhibited a slight weight loss. However, there was no significant difference in weight changes between the control group and those treated with either drug (Figure 5B). Remarkably, lung viral titers in mice treated with thioinosinic acid at a dosage of 5 mg/kg showed a substantial reduction compared to the vehicle control group. The viral load in these mice was approximately tenfold lower than that in the vehicle-treated group (Figure 5C). By contrast, treatment with azathioprine at the same dosage did not demonstrate a significant reduction in viral titers compared to the control group. This outcome might be attributed to the pharmacodynamic properties of azathioprine, suggesting a differential efficacy profile between the two drugs.

## 4. Discussion

This study leveraged a hybrid computational–experimental approach to unearth potent inhibitors of SARS-CoV-2. Our efforts led to the identification of 19 compounds that effectively curbed virus infection in vitro, with eight exhibiting broad-spectrum activity across diverse cell lines such as Caco-2 and Vero cells. A noteworthy discovery is the antiviral efficacy of azathioprine, an FDA-approved immunosuppressant, against SARS-CoV-2. Its active metabolite, thioguanosine triphosphate (TGTP), integrates into DNA and RNA during cell division, disrupting the proliferation of rapidly dividing cells, including lymphocytes. This mechanism suggests that TGTP might also incorporate into the rapidly replicating RNA of SARS-CoV-2, potentially inducing mutations that hinder viral replication [52]. 

Our findings are particularly significant when compared to existing COVID-19 treatments such as remdesivir, molnupiravir, and the nirmatrelvir–ritonavir combination (Paxlovid). While remdesivir can shorten recovery times, its high cost and intravenous administration limit its utility for mild cases. Molnupiravir shows promise in reducing hospitalization risk in high-risk individuals but is less effective when administered later in disease progression. Paxlovid significantly lowers hospitalization or death risk but poses challenges in cost and potential drug–drug interactions, especially in patients with chronic health conditions [53]. By contrast, azathioprine, with its antiviral and anti-inflammatory properties, emerges as a promising candidate. It could potentially mitigate severe immunological responses, such as cytokine storms and hyperimmune activation, which are observed in advanced COVID-19 stages. The existing FDA approval of azathioprine accelerates its potential integration into clinical trials, either as a standalone treatment or in combination with other antivirals.

While advanced drug discovery methods like molecular docking and molecular dynamics simulations offer insightful predictions, they have inherent limitations. Molecular docking often uses fixed protein structures, failing to represent their dynamic physiological state. Molecular dynamics simulations, though detailed, require extensive computational resources and capture only short timescales, missing broader structural transitions. Artificial intelligence algorithms can expedite the prediction of new compound activities but are constrained by the need for large, high-quality datasets of established active compounds.

Our computational screening identified virus inhibitors using data primarily from Vero cell screenings. To enhance translation success, future efforts should develop comprehensive training data pipelines incorporating diverse cell screenings. This strategy can help avoid compounds active only in specific cell lines, widening the search for effective inhibitors.

Experimentally, several critical questions remain. Identifying the molecular targets of the initial hit compounds and evaluating their antiviral efficacy in human primary cells are essential next steps to validate their clinical relevance. Furthermore, while azathioprine showed limited inhibition in mouse models, additional studies on dosage and administration are warranted to fully assess its therapeutic potential.

In conclusion, our study underscores the potent synergy between computational and experimental methods in identifying antivirals against SARS-CoV-2. This integrated approach has expedited the discovery of promising therapeutic candidates, marking a significant stride in the ongoing battle against the virus.

## Figures and Tables

**Table 1:** The activity and cytotoxicity of active compounds.

Compound ID	EC50 (µM)	CC50 (µM)
C3	1 ± 2.1	21.18
C5	1 ± 1.9	>50
C7	3.9 ± 1.8	>50
C8	1.5 ± 1.7	>50
C9	6.6 ± 1.8	>50
C10	5.3 ± 1.7	>50
C11	6.7 ± 2.1	>50
C19	1.7 ± 2	>50

**Table 2:** Potential biological activities of the hit drugs.

Compound ID	EC50 (µM)	Predicted Targets
C3	1.0	Atypical chemokine receptor 3
C5	1.0	Estrogen-related receptor gamma
C8	1.5	Low molecular weight phosphotyrosine protein phosphatase
C19	1.7	Retinal rod rhodopsin-sensitive cGMP 3’,5’-cyclic phosphodiesterase subunit delta
C7	3.9	Adenosine kinase
C10	5.3	Urotensin-2 receptor
C9	6.6	Potassium/sodium hyperpolarization-activated cyclic nucleotide-gated channel 1
C11	6.7	Sigma intracellular receptor 2

Table showing hit compounds EC_50_ values and their predicted targets based on similarity prediction analysis.

## Data Availability

The custom python scripts that the deep learning model implements are available on GitHub: https://github.com/midoabdo14/Deep-Inhibitors. (accessed on 1 October 2023). This repository also includes all associated files and data needed to execute the scripts.

## Supplementary Materials

The following supporting information can be downloaded at: https://www.mdpi.com/article/10.3390/biomedicines11123134/s1, Figure S1. Data to train SARS-CoV-2 drugs classification model. A: The preparation of preliminary data, showing the proportion of effective drugs (100 drugs) to ineffective ones (2000 drugs). B: Box plot shows the size distribution of compounds within the training dataset. The number of atoms present in the active and non-active subsets were calculated and plotted with an RDkit and matplotlib libraries. Figure S2. Structures and sources of the potential SARS-CoV-2 inhibitors. This collection comprises compounds that are commercially accessible, as well as those provided to KCB by independent researchers. Figure S3. Thioinosinic acid inhibits in vitro SARS-CoV-2 infection. A: The dose-dependent inhibition of SARS-CoV-2 by thioinosinic acid in the Calu-3 cell line. The cells were infected with the virus one hour prior to treatment, and then the drug was added at twofold dilutions for 72 h. A plaque assay was used to measure the infectious virus titer in cell supernatants. The error bars represent the mean ± SD of duplicate samples. Statistical analysis was performed using one-way ANOVA with Dunnett’s test, with *** *p* < 0.0002. B: Drug cytotoxicity in cells without infection was measured and normalized to DMSO-treated cells. Figure S4. In Vitro Inhibition of SARS-CoV-2 Infection by Thioinosinic Acid. A. Illustrates the effect of thioinosinic acid on SARS-CoV-2 in the Calu-3 cell line. The cells were first infected with the virus at an MOI of 0.1. One hour after infection, thioinosinic acid was administered in two-fold dilutions, and the treatment continued for 72 h. The infectious virus titers in the cell supernatants were quantified using a plaque assay. The error bars represent the mean ± standard deviation (SD) of duplicate samples. Statistical significance was evaluated using one-way ANOVA with Dunnett’s test, indicated by *** *p* < 0.0002. B. The cytotoxicity of thioinosinic acid in uninfected Calu-3 cells was measured. The results were normalized against cells treated with DMSO to assess the drug’s safety profile.
