# Supplementary material for: How Deep Learning in Antiviral Molecular Profiling Identified Anti-SARS-CoV-2 Inhibitors
Source: Biomedicines. 2023 Nov 24;11(12):3134. doi: 10.3390/biomedicines11123134 (PMC10740425; doi:10.3390/biomedicines11123134)
Supplement: Supplementary file 1 [file biomedicines-11-03134-s001.zip › biomedicines-2682245-supplementary.pdf]

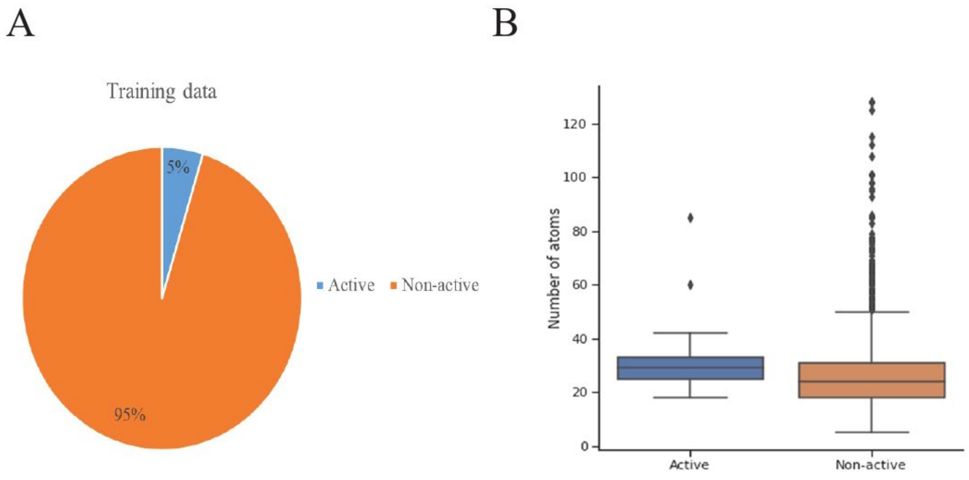

Figure S1. Data Preparation for Training the SARS-CoV-2 Drug Classification Model.

A. Illustrates the initial composition of the dataset used to train the classification model. It shows the proportion of effective drugs (100 drugs) compared to ineffective ones (2000 drugs).

B. Box plot shows size distribution of compounds within the training dataset. The number of atoms present in the active and non-active subsets were calculated and plotted by RDkit and matplotlib libraries.

C3 Compound

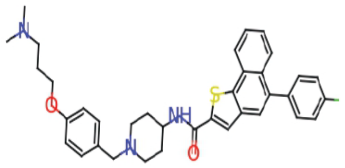

C5 Compound

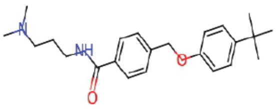

C7 Compound

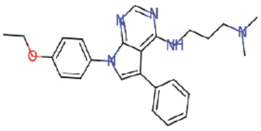

C8 Compound

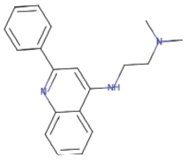

C9 Compound

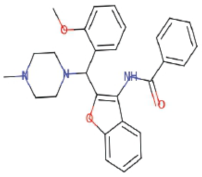

C10 Compound

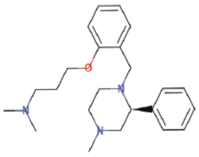

C11 Compound

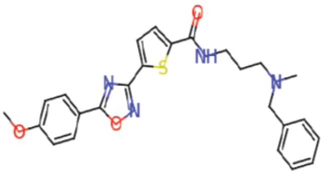

C19 Compound

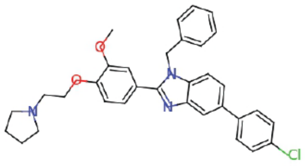

Figure S2. Structures and Sources of Potential SARS-CoV-2 Inhibitors.

The collection of compounds identified as potential inhibitors of SARS-CoV-2. It includes both commercially available compounds and those contributed by independent researchers to the Korea Chemical Bank (KCB).

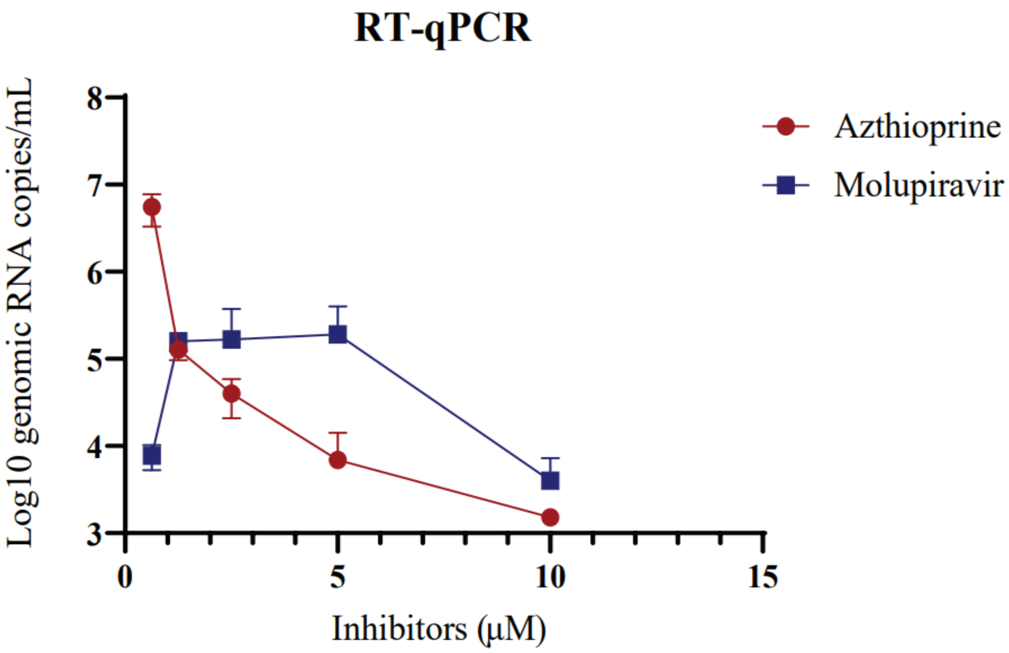

Figure S3. In Vitro Inhibition of SARS-CoV-2 Infection by Azathioprine

This figure illustrates the dose-dependent inhibition of SARS-CoV-2 by azathioprine in the Calu-3 cell line. An RT-PCR assay was employed to quantify the infectious virus titer in the presence of varying concentrations of azathioprine. The graph displays the results of this quantitative analysis, with error bars indicating the mean ± standard deviation (SD) of duplicate samples.

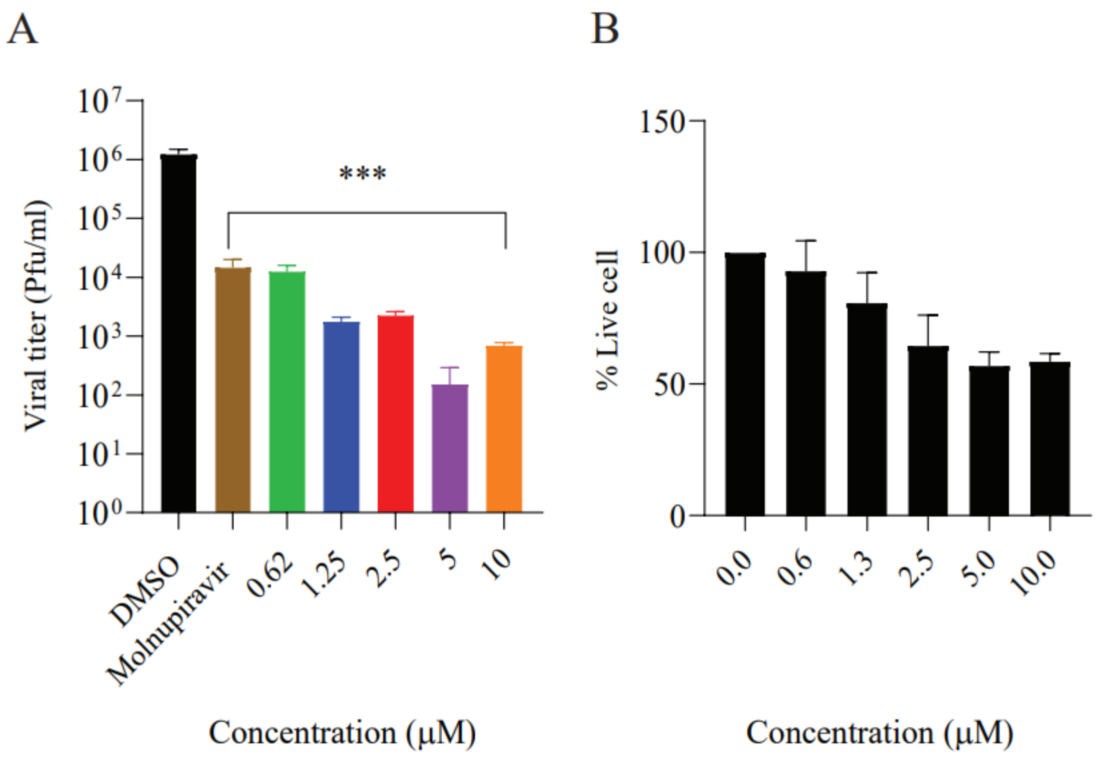

Figure S4. In Vitro Inhibition of SARS-CoV-2 Infection by Thioinosinic Acid.

A. Illustrates the effect of thioinosinic acid on SARS-CoV-2 in the Calu-3 cell line. The cells were first infected with the virus at an MOI of 0.1. One hour after infection, thioinosinic acid was administered in two-fold dilutions, and the treatment continued for 72 hours. The infectious virus titers in the cell supernatants were quantified using a plaque assay. The error bars represent the mean ± standard deviation (SD) of duplicate samples. Statistical significance was evaluated using one-way ANOVA with Dunnett's test, indicated by ***$p < 0.0002$.

B. The cytotoxicity of thioinosinic acid in uninfected Calu-3 cells was measured. The results were normalized against cells treated with DMSO to assess the drug's safety profile.
